# Unraveling Tumor Heterogeneity in an Apparently Monolithic Disease: BCL2 and Other Players in the Genetic Landscape of Nodal Follicular Lymphoma

**DOI:** 10.3389/fmed.2019.00044

**Published:** 2019-03-15

**Authors:** Francesca Magnoli, Maria Grazia Tibiletti, Silvia Uccella

**Affiliations:** ^1^Unit of Pathology, Department of Medicine and Surgery, University of Insubria, Varese, Italy; ^2^Department of Pathology, ASST Sette Laghi, Varese, Italy

**Keywords:** follicular lymphoma, clonal evolution, clonal heterogeneity, cytogenetics, translocation *t*(14;18)(q32;q21), somatic hypermutation

## Abstract

Follicular lymphoma (FL) is the most common form of non-Hodgkin lymphoma in Western countries. Although traditionally considered a well-defined, easy to diagnose lymphoproliferative disorder, in the last few years it has become clear that it is in fact composed of many different clinicopathological entities, encompassing a variegated and complex genetic background. This has led to the inclusion of specific FL variants and separate entities in the latest update of the WHO classification. However, even in the context of classical FL, many aspects of intra- and inter-tumoral heterogeneity have been recognized, with a major influence on diagnosis and clinical practice at different time points during the course of the disease. This review focuses on the molecular cytogenetic heterogeneity in classical FL from precursors and early development to progression and transformation, in terms of both clonal heterogeneity and unusual genetic features. Several factors have been investigated and suggested to contribute to the broad spectrum of clinicopathological, phenotypic, and genetic features observed in otherwise morphologically classical cases. Among them, deregulation of the epigenetic machinery and interactions with tumor microenvironment seem to play a pivotal role, together with genetic aberrations involving well-known molecular pathways and mechanisms physiologically operating in the germinal center. In the era of personalized medicine, precision diagnostics based both on understanding of the complex interplay among all these factors and on novel developments will become crucial to predict the outcome and guide the treatment of FL patients.

## Introduction

Follicular lymphoma (FL) is the most common form of non-Hodgkin lymphoma in Western countries. It is defined as a neoplasm composed of germinal center (GC) B cells (typically both centrocytes and centroblasts) with at least a focal follicular growth pattern (1). In fact, such a broad definition, rather than identifying a monolithic entity, encompasses a spectrum of different morphologic, immunophenotypic, genetic, and clinical pictures, which make FL a very complex and heterogeneous disease. In the last decade, a growing burden of evidence has been supporting the concept that this heterogeneity, if correctly recognized, rather than being an obstacle to the management of the patient, can represent the start point for a tailored treatment. Indeed, depending on the disease's features, some forms of FL may require no systemic therapy at all, others may benefit of chemo-free regimens based on monoclonal antibodies and small molecules, whereas aggressive chemotherapy can be needed in a subset of FL.

Firstly, the heterogeneity of FL is morphologically reflected by the fact that it is the only non-Hodgkin lymphoma that can be subdivided, based on the number of centroblasts per high-power field (HPF), into low grade (grade 1–2), and high grade (grade 3A and 3B) forms. This underlies important differences in the biological and clinical behavior of the neoplasm, that imply different management of the patients. The combination of the grade and the pattern of growth (follicular, diffuse or mixed, follicular, and diffuse) heavily influences the final diagnosis, in that diffuse areas with more than 15 centroblasts per HPF should be reported as diffuse large B-cell lymphoma (DLBCL), and not as grade 3A FL, as it would be in a follicular pattern of growth (1). Interestingly, the transition from low grade through high grade forms to DLBCL is part of the natural history of most FLs, and it puts FL heterogeneity both in a spatial (different grades of the disease in the same lymph node or synchronously at different sites) and in a temporal (morphological changes in sequential biopsies in the same patient) perspective. Also the immunophenotype of FL cell, with the expression of the germinal center markers CD10 and BCL6 considered as the hallmark of the disease, has been demonstrated not to be so constant. Actually, a subset of cases lack one (usually CD10) or both of the markers and this has risen the need for additional immunohistochemical tools for the recognition of the disease. Another hot topic in the immunophenotyping of FL is BCL2 expression. The positivity of the germinal center cells for this marker has been long regarded to as pathognomonic of FL. However, a subset of cases has been found not to express this marker, or to present only a faint and inhomogeneous staining, also when tested with multiple monoclonal antibodies directed against different epitopes of the protein, such as 100/D5, E17, SP66 (2, 3). Thus, it is now clear that neither BCL2-positivity is diagnostic for FL, nor BCL2-negativity excludes this type of lymphoma. The heterogenous pattern of BCL2 expression in FL mirrors the variable presence and types of *BCL2* gene abnormalities in tumor cells. Indeed, the role of the translocation *t*(14;18)(q32;q21), involving *BCL2* and causing the hyperexpression of the antiapoptotic protein, which has long been considered to drive the neoplastic proliferation and to represent the cytogenetic marker of the disease, has been revised in the last few years and novel genetic data on the disease have emerged (see below in the text). Finally, from a clinical point of view, the biological heterogeneity of FL is reflected in the wide spectrum of clinical presentations (in terms of patient's age, site of insurgence, and extent of the disease at diagnosis) and disease behavior (from indolent to aggressive forms).

The above mentioned heterogeneity of the disease has been at least partly acknowledged by the inclusion of four clinicopathological variants of FL in the latest update of the WHO classification, namely *in situ* follicular neoplasm, duodenal-type FL, testicular FL, ad diffuse FL. In addition, separate entities, such as pediatric-type FL, large B-cell lymphoma with *IRF4* rearrangement, and primary cutaneous follicle center lymphoma have been recognized. However, besides these well-defined entities, other aspects of the inter- and intra-tumor heterogeneity of FL are evident. In particular, the analysis of the genetic profile of tumor cells highlights important relationships between specific genetic lesions and tumor initiation, progression, and transformation.

This review will focus on the molecular cytogenetic heterogeneity in nodal FL, without discussing the clinicopathological variants and separate entities. In particular, we will concentrate on the growing genetic complexity of the disease, from precursors and early development to progression and transformation, in terms of both clonal heterogeneity and unusual genetic features, with a special focus on *BCL2* alterations.

## From Early FL Development to Progression and Transformation

**Figure 1:**
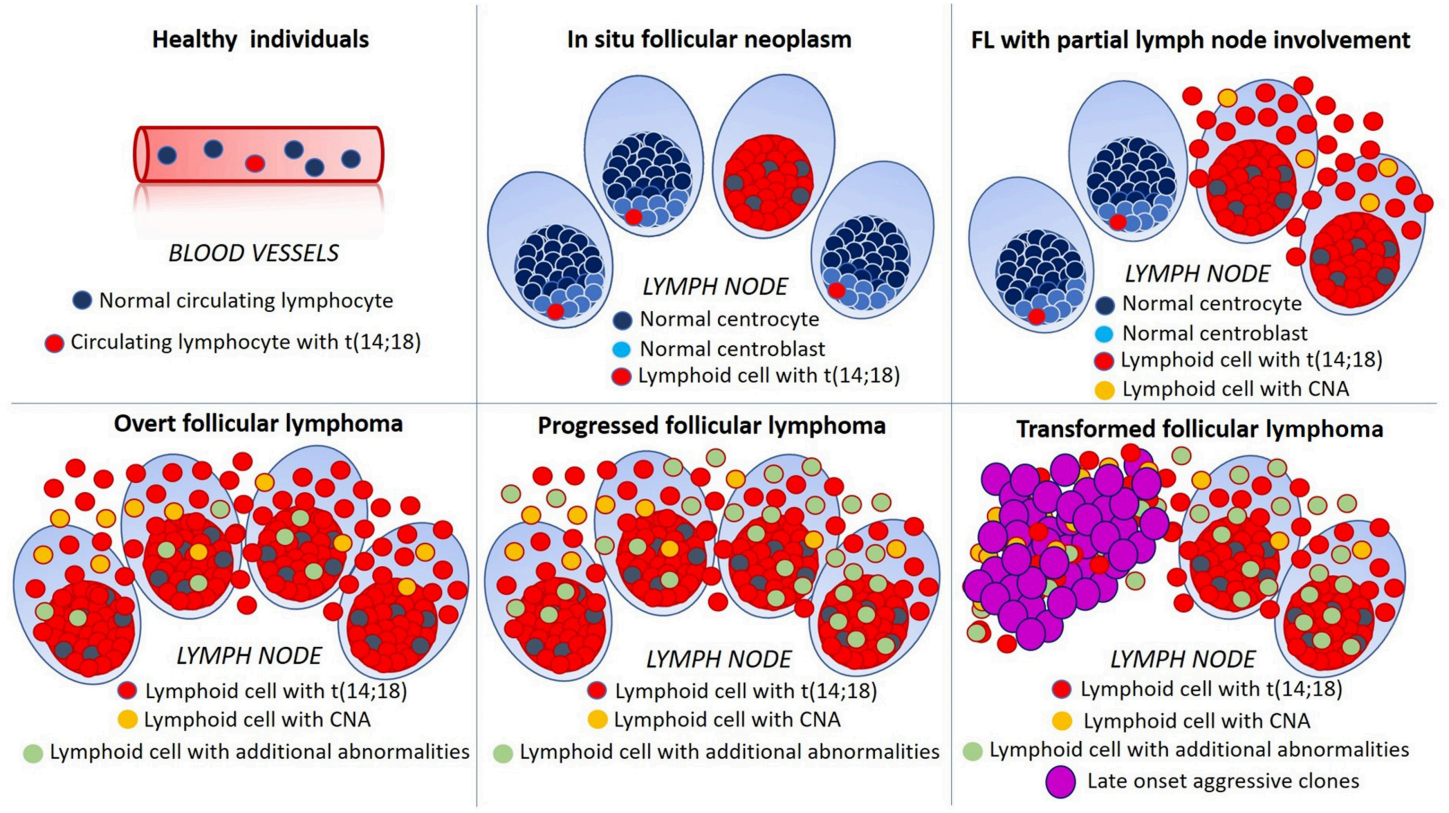
Schematic representation of multistep lymphomagenesis, progression and transformation in follicular lymphoma (FL). In 70% of healthy individuals, circulating lymphocytes bearing *t*(14;18)(q32;q21) can be detected albeit, in the majority of cases, they never progress to FL. *In situ* follicular neoplasms is defined by the presence of *t*(14;18)(q32;q21) in germinal center cells of architecturally normal lymph nodes. In early FL with partial involvement of the lymph node, neoplastic translocated cells are present also outside germinal centers and efface nodal tissue. In overt FL additional genetic abnormalities, such as chromosomal gains and losses (copy number alterations, CNA) become evident and, with progression their number and complexity increase. The emergence of aggressive clones, bearing multiple genetic alterations, is mirrored by morphological changes, such as the diffuse growth of large lymphoid cells and dramatically worsens patient's outcome.

In recent years, it has been recognized that FL lymphomagenesis is a multistep process, in which early lesions progress to overt disease through complex events of selection/counter-selection (Figure 1). Using an exome sequencing approach, Green and coworkers proposed an elegant genetic evolution model for FL tumorigenesis in which founder mutations [such as *t*(14;18)] turn a non-malignant B cell clone into a premalignant tumor cell population, stable enough to acquire one or more secondary driver mutations (such as *CREBBP*), leading to an early malignant clone. Finally, tertiary mutations (such as *MLL2* and *TNFRSF14*) may either act as passenger or accelerator mutations, the latter providing a selective advantage to a progressed malignant subclone (4).

## Early Steps in Lymphomagenesis

At the beginning of this spectrum, the earliest known oncogenic event is the *t*(14;18)(q32;q21). It seems to occur in bone marrow immature pre-B cells, due to erroneous V(D)J recombination, and results in constitutive expression of the BCL2 antiapoptotic protein. Under normal conditions, upon antigen challenge, GC B cells undergo proliferation, somatic hypermutation, and antigen-affinity selection, so that only those with high B-cell receptor (BCR) affinity will survive and further differentiate. In this scenario, down-modulation of BCL2 expression is necessary to render GC B cells highly sensitive to apoptosis, in order to eliminate potentially dangerous autoreactive or low-affinity elements. In *t*(14;18)+ B cells, this pro-apoptotic program clearly doesn't work, and, after entering the GC, they can survive irrespective of their BCR affinity with the risk of oncogenic transformation. Surprisingly, it has been demonstrated that both naïve and antigen-experienced *t*(14;18)+ B cells can be detected at low levels in the peripheral blood of up to 70% of healthy adults, most of which will never develop overt FL (5, 6). Thus, the hallmark *t*(14;18)(q32;q21) has been reinterpreted as possibly necessary, although not sufficient to develop a fully malignant phenotype. In this context, *BCL2* deregulation provides a survival advantage that might favor the acquisition of additional genetic aberrations during repeated transits of BCL2-overexpressing B cells through the GC (7).

Another early step in FL development is possibly related to the acquisition of asparagine (N)-linked glycosylation sites in the Ig variable regions through somatic hypermutation (SHM) (8). This event, which is characteristic of FL, albeit it is also observed in other B-cell lymphomas, may explain the retention of BCR activity in neoplastic cells. Actually, although *t*(14;18)(q32;q21) disrupts one Ig allele, the expression of surface B cell receptor (BCR) is retained in most FL cells. In fact, expression of surface Ig and maintenance of its signaling activity even in the absence of antigen is mandatory for normal B-cell survival and has been shown to be crucial for the majority of B-cell malignancies. Indeed, the retention of BCR signaling may be mediated by acquisition of asparagine (N)-linked glycosylation sites in the Ig variable regions. In contrast to germ-line-encoded glycosylation sites in the constant BCR region, these altered variable region glycosylation sites carry mannose-terminating sugars, suggesting a potentially important interaction of FL cells with mannose-binding lectins of the innate immune system in the germinal center. In brief, high-mannose glycans in surface Ig may represent the mechanism by which surface Ig activate the malignant cells even in the absence of antigen, hence promoting tumor progression (9–11). Candidate molecules for this interaction include C-type lectins expressed by macrophages and dendritic cells, which have been proposed as possible targets for new therapeutic strategies. In this regard, promising agents include both antibodies against high-mannose glycans and mannose-based oligosaccharide mimics or non-carbohydrate glycomimetics that act as competitive inhibitors of lectin–glycoprotein interactions are currently being developed against human immunodeficiency virus, since envelope glycans of this virus are almost entirely of the oligomannose type (12). Further studies are needed to validate the efficacy of these therapeutics in the treatment of FL.

## *In situ* Follicular Neoplasia

Among putative precursors of FL, *in situ* follicular neoplasia (ISFN) has been first described in 2002 by Cong et al. (13), as the presence of occasional follicles containing clonal, BCL2 brightly positive abnormal B cells in an otherwise architecturally and cytologically normal lymph node. Even if ISFN has been proposed as the tissue counterpart of *t*(14;18)+ B cells seeding sparse GCs (14), it is now clear that it represents a clonal lesion carrying additional genomic alterations (15). In detail, high resolution comparative genomic hybridization (CGH) array documented low levels of copy number alterations (CNA) in terms of gains rather than losses, with frequent involvement of chromosomes 1 and 18. Among amplified genes, only a few could be recognized as recurrent and potentially relevant in FL lymphomagenesis, and besides known oncogenes (such as *BCL2, RUNX1*, and *KDSR*), they interestingly include genes functionally related to the biology of the GC, namely *TOX, BACH2, AFF3*, and *EBF1*. Finally, mutations in histone-modification genes, such as *CREBBP* and *EZH2* have been detected in ISFN as early driving events (4, 16).

## Partial Involvement of Lymph Node by FL

**Figure 2:**
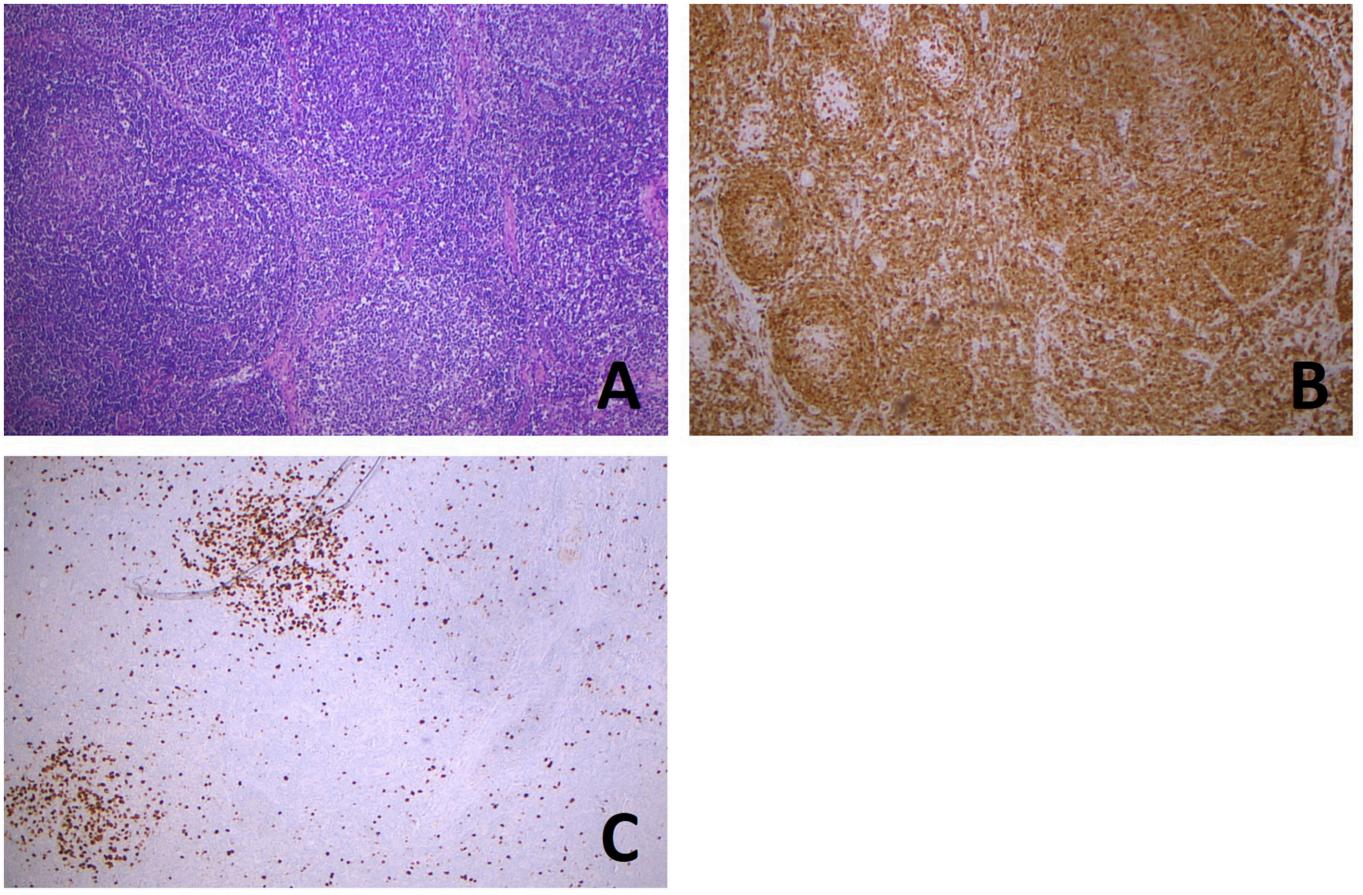
Partial involvement of lymph node by FL. Hematoxylin and eosin stain **(A)** shows the coexistence, in the same lymph node slide, of normal lymphoid follicles (left), with well-defined mantels and hyperplastic germinal centers, with a low grade follicular lymphoma (right) showing irregularly shaped and confluent neoplastic follicles. BCL2 immunostaining **(B)** is negative in hyperplastic germinal centers (left) and positive in a large neoplastic center (right). Ki67 proliferative index **(C)** is high in hyperplastic centers (left and top), whereas in the neoplastic proliferation it is very low (right and bottom). (All images, original magnification x50).

ISFN must be distinguished from partial involvement by FL (PFL) (Figure 2), which is characterized by altered node architecture with some residual reactive follicles (14). Although patients with PFL usually present with low stage disease, they seem to be at higher risk of progression to overt FL than ISFN (approximately 50 vs. 5%, respectively) (14, 16). In line with this clinical observation, PFL represents a more genetically advanced lesion in the spectrum of FL precursors, in terms of both mean number and size of alterations detected at array CGH analysis. Among copy number alterations (CNA), gains were prevalent, but not exclusive as in ISFN. Intriguingly, as high as 99% of them was shared with overt FL, suggesting that significant selective pressure is already acting at PFL level. Amplifications involve known oncogenes as well as histone modifiers (*EZH2, MLL2, ARID2, HDAC7*), again underlying the importance of deregulated chromatin biology in lymphomagenesis. By contrast, most of recorded deletions probably represent passenger alterations, as <20% of them were shared with manifest FL. Affected targets notably include tumor suppressor genes (for example *TP73*) and genes mapping on 1p, such as *TNFRSF9* and *TNFRSF14*, a member of the tumor necrosis factor receptor superfamily, that has emerged as one the most frequent acquired genetic aberration in FL (17).

## Progression and Transformation

Progression to overt FL is associated with a statistically significant increase in the mean number of genomic aberrations per sample at array CGH. On the contrary, the mean size of alterations expressed in base pairs does not differ among ISFN, PFL, low grade, and grade 3A FL, even if large alterations (>10 Mb) are rarely observed at the beginning of the spectrum in ISFN (18). The aforementioned hierarchical model of FL lymphomagenesis proposed by Green et al. (4) outlines a strong tumoral dependency on deregulating epigenetic events in the course of the disease. Co-occurring aberrations of genes involved in B-cell development, JAK-STAT and NF-κB signaling, as well as interactions with tumor microenvironment play a major role in the genesis, progression, and transformation of FL (19).

**Figure 3:**
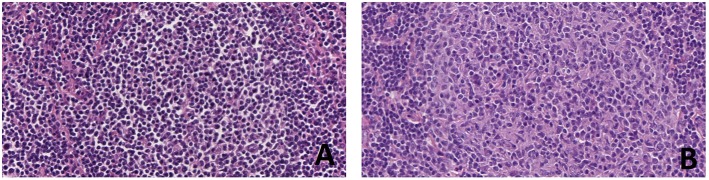
Progression of follicular lymphoma in sequential lymph node biopsies in the same patient. At first diagnosis **(A)** the patient had a low grade (grade 1–2 of 3) FL with a simple karyotype, only showing *t*(14;18)(q32;q21). After 3 years, the FL recurred as a grade 3A **(B)** and a complex karyotype (81-84XXYY,-Y,-Y,-2,-3,del(3)(p21pter),der(3),*t*(14;18)(q32;q21)ish,*t*(14;18)(Igh+,Bcl2+;Igh+,Bcl2+),-7,-10,*t*(14;18)(q32;q21)ish,*t*(14;18)(Igh+,Bcl2+;Igh+,Bcl2+)+mar (personal data, unpublished) (H&E, original magnification x400).

**Figure 4:**
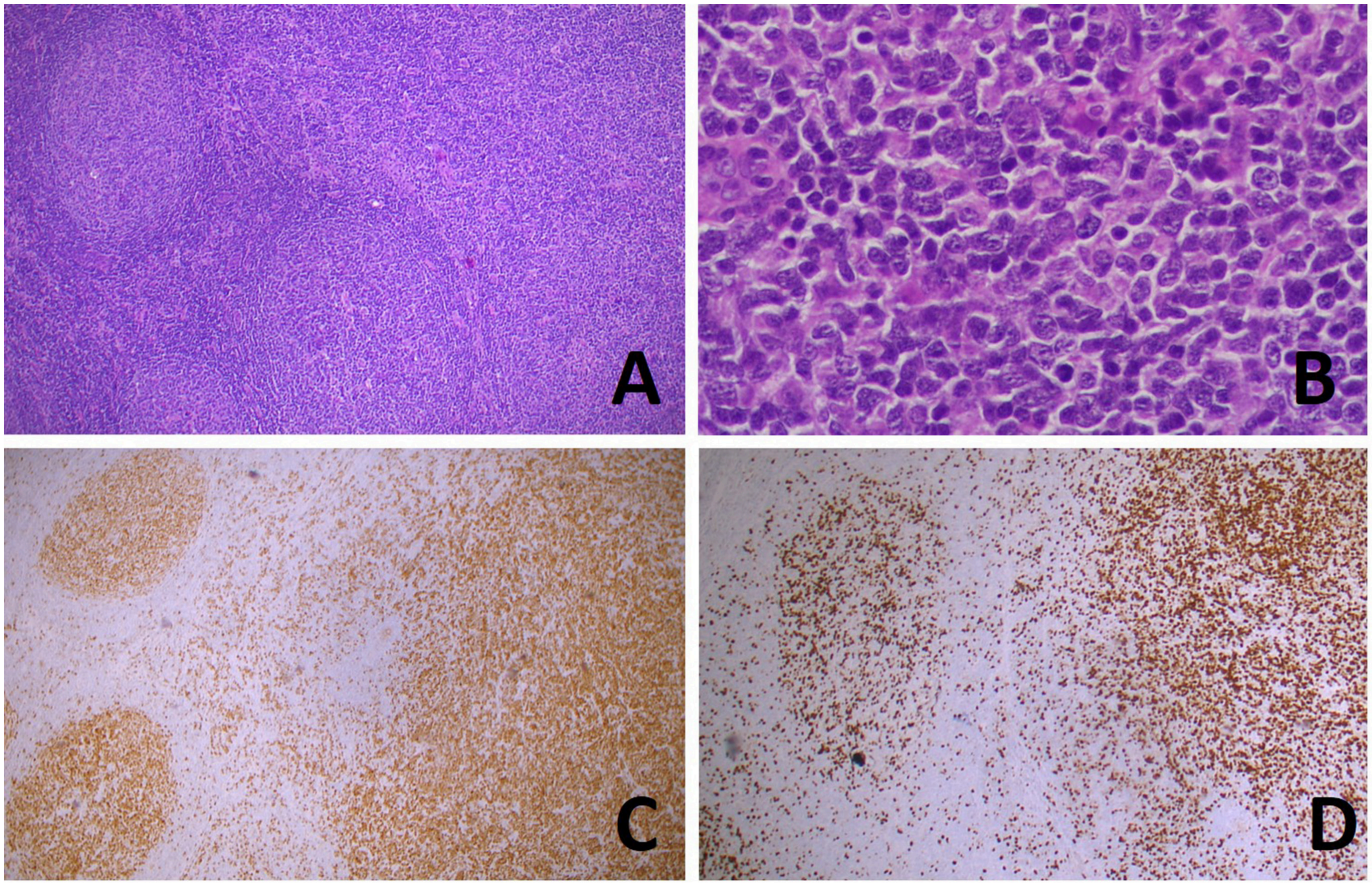
Transformation of follicular lymphoma (FL) in a diffuse large B-cell lymphoma (DLBCL). Hematoxylin and eosin stain **(A)** shows the coexistence, in the same lymph node slide, of FL (left) and DLBCL [right, higher magnification in **(B)**]. BCL6 **(C)** and Ki67 **(D)** immunostainings highlight, respectively, the nodular and diffuse growth of the two components). [**(A,C,D)**: original magnification x50; **(B)**: original magnification x400].

Early progression after immunochemotherapy, as well as morphological transformation to a more aggressive lymphoproliferative disorder (grade 3 FL or diffuse large B-cell lymphoma, see Figures 3, 4) are both associated with a poor effect on prognosis (20–23). It is now clear that such dismal events are not driven by a single hallmark genetic alteration, but rather by distinct aberrations which may also be acquired early on, even if their adverse impact may become evident only at more advanced stages or under therapeutic pressure. Shedding light on the nature of FL evolutional dynamics may operatively translate into more effective diagnostic and therapeutic strategies. In this regard, different modalities of evolution have been proposed for progressed (PRFL) and transformed FL (TFL), the latter being characterized by the late emergence of aggressive clones, whereas the former resulting from prevalent clones already demonstrable at diagnosis (24). Analyzing sequential disease samples by array CGH, some authors documented a trend for increasing genomic complexity in the transition from 1–2 low grade to 3a FL, in terms of both gains of chromosome 5, 8q24, 11p11, 12q, 16, 18, 21, and Xp and losses involving 6q and 17p. However, the overall CNA-frequencies remained relatively stable throughout the course of the disease, as in the majority of the cases one or more of the CNAs present in the initial samples were absent in the late samples, where new CNAs were acquired (25). Intriguingly, mutations of selected peculiar genes have been associated with early progression, including *KMT2C, TP53, BTG1, MKI67, XBP1*, and *SOCS1* (17). None of these genes is included in the m7-FLIPI clinico-genetic risk index, recently developed to improve outcome prediction for patients requiring immunochemotherapy (26), suggesting the need of even more precise prognostic tools. Mechanisms underlying FL transformation to DLBCL have been studied for years in small series with a candidate-gene approach, with the identification of several genetic, epigenetic, and microenvironmental factors playing a role in the biology of such unfavorable event. Only in recent years, further investigation by whole exome sequencing and copy number analyses, allowed to demonstrate that FL transformation does not represent a linear process, in which the transformed dominant clone originates directly from the initial FL dominant population through the sequential acquisition of additional alterations, but results from the divergent evolution of a common precursor cell that acquires distinct genetic lesions to become a TFL. In this model, commonly shared lesions occurring early in the ancestral precursor have been identified, including deregulation of pathways involved in chromatin regulation and apoptosis, whereas TFL is specifically associated with *CDKN2A/B, TP53*, and *MYC* alterations, leading to impairment of cell cycle progression and DNA damage responses, as well as aberrant somatic hypermutation (see next paragraph), targeting several genes including *PIM1, PAX5, RhoH/TTF, MYC, BCL6, BCL7A, CIITA*, and *SOCS1* (27, 28).

## Clonal Heterogeneity in FL

Clonal heterogeneity can be defined as the presence of several subclones harboring different genomic aberrations within a specific neoplasm. This phenomenon may result from genetic and epigenetic alterations, interaction with microenvironmental factors, as well as variation in cellular function both stochastic and therapy-induced. Cooperation among different subclones may provide tumor growth advantage and therapeutic resistance, and as such represents a potentially target to inhibit disease progression (29).

As already stated, *t*(14;18)(q32;q21) juxtaposing the *BCL2* gene to the *IGH* 3′ regulatory regions (RRs) represents the hallmark genetic event defining FL. However, significant inter- and intra-tumoral heterogeneity of BCL2 protein expression among translocated cases and differential sensitivity to therapy of distinct sub-populations of FL cells in individual patients have been demonstrated (30). In the same study, overall survival was significantly higher in cases expressing lower BCL2 levels, remarking the importance of inter-tumoral heterogeneity, even though when performing multivariate analysis using the significant parameters emerging from the univariate analysis, the only independent prognostic factor for disease-free survival and overall survival was the prognostic index FLIPI-2 (30).

An important player in clonal heterogeneity of FL is represented by activation-induced cytidine deaminase (AID), a gene whose product is required in the highly specialized germinal center microenvironment for both SHM and class switch recombination (CSR) to generate high affinity antibodies (31). Although predominantly restricted to immunoglobulin genes, aberrant AID activity has been shown to target additional loci, mostly with the properties of super-enhancer domains (32). These are composed of large arrays of interconnected promoters and enhancers, that display unusually high levels of transcription and epigenetic accessibility (33). Mutations are variably distributed in the 5′ untranslated or coding sequences, and share features typical of V region associated SHM (34). Moreover, off-target deamination recruits base excision repair (BER) and mismatch repair (MMR) machineries, which create nicks and double-strand breaks, favoring chromosomal translocations. Thus, abnormal functioning of the physiological SHM process may lead to genetic instability and drive clonal heterogeneity in FL. Finally, as already stated, AID represents a major contributor to transformation to DLBCL, favoring the acquisition of additional driver mutations (35). Intriguingly, some authors have reported an intracellular mechanism by which Epstein-Barr virus (EBV)-encoded latent membrane protein-1 (LMP1) induces AID upregulation via the transcription factor Egr-1 (36). EBV-positive FL is an uncommon disease, observed with a prevalence of approximately 2.5% in the largest published series (37, 38). In the first report by Mackrides and coworkers, all EBV-positive cases in which biopsy material was available at separate time points (7 out of a total of 10 EBV-positive FL) demonstrated progression of the disease to a higher-grade FL or to DLBCL (37). The same authors did not confirm this observation in a larger series of 488 unselected FL, where none of the 12 patients with EBV-positive FL experienced transformation to DLBCL. However, they showed a trend toward more aggressive clinical features, including higher FLIPI score (38). In summary, current data suggest that, in a restricted subset of FL, EBV infection may have a role in both lymphomagenesis and disease progression/transformation, by promoting genomic instability through AID expression. Further investigations are needed to clarify the impact of EBV status on clinical course as well.

As clonal heterogeneity emerged as an independent predictor of poor prognosis in DLBCL and mantle cell lymphoma, it could be useful to integrate it with classical prognostic biomarkers in order to improve the prediction of patients' clinical outcome. However, its incidence in FL has been correlated with disease stage but not with differences in survival (39). By using a combination of whole exome and targeted deep sequencing, Araf and coworkers analyzed a cohort of nine FL patients, collecting for each two spatially separated synchronous biopsies. Intratumor heterogeneity (ITH) was expressed by a coefficient representing the ratio of shared to total (shared and discordant) genetic variants for two paired samples, with obtained values ranging from 0.92 (greatest similarity) to 0.41 (lowest similarity). Again, higher levels of ITH did not translate into a more adverse outcome, but these results raise the important question that evaluation of a single biopsy is not able to adequately capture a patient's genetic heterogeneity and it may preclude the administration of target therapies due to failure in the detection of the corresponding predictive biomarker in the analyzed sample (28). Besides concordant involvement of different sites, this spatial form of ITH may manifest as discordant lymphomas in patients simultaneously diagnosed with a high-grade and a low-grade lymphoma typically involving lymph nodes and bone marrow, respectively. Another approach to define ITH was employed by Spence and coworkers, who performed an ultra-deep sequencing analysis to demonstrate that the amount of aberrant SHM at non-IGH sites may be well-estimated from aberrant SHM of BCL2 locus, whereas there seems to be no relationship between the entity of aberrant and physiological SHM. These authors concluded that aberrant SHM represents a valid tool to quantify intra-tumoral heterogeneity (ITH) (40). Its association with recurrent CNAs involving oncogenes and tumor suppressor genes including *MYC, CDKN2A/B*, and *TP53*, suggests that deregulation of proliferation, apoptosis and cell cycle may play a pivotal role in clonal heterogeneity.

Another example of ITH is represented by composite lymphomas, consisting of at least two different entities that occur simultaneously in the same organ as a collision tumor. From a morphological point of view, tumor borders can be sharply defined, or tumor cells can be variously intermixed. Molecular studies have demonstrated that the malignant clones develop separately from a common altered precursor, usually of germinal center derivation, after acquiring additional separate transforming events (41).

Since the seminal study of gene expression profile (GEP) analysis by Dave and coworkers, who identified two different immune-response gene signatures in a cohort of untreated FL, the composition of tumor microenvironment has been demonstrated to be relevant in shaping the biology and clinical behavior of FL, and is also involved in ITH (42). Indeed, the cross-talk between lymphoma and immune cells is involved both in supporting tumor growth and survival, including clone selection, and in suppressing the antitumoral immune response. Even if studies investigating the prognostic impact of the composition of tumor microenvironment are highly conflicting (43–47), there is growing evidence of the importance of understanding the microenvironment as the rationale to employ new therapeutics targeting the immune system in the treatment of FL. However, as this review focuses on the intrinsic genetic alterations of FL neoplastic cells, the reader is addressed to specific reviews on this interesting argument (48, 49). In this context, it is only worth to add that also therapeutic agents may induce changes in FL microenvironment, further promoting ITH. Actually, besides eliminating tumor cells, cytotoxic agents are also responsible of collateral events, both in surviving neoplastic cells, such as genomic instability and the development of a senescent-associated secretory phenotype (SASP), and in the microenvironment, by promoting chronic inflammation, hypoxia and wound healing responses (29).

## Unusual Genetic Features in FL

As previously stated, the translocation (14;18)(q32;q21) is considered the genetic hallmark of FL and is reported with a prevalence of 85–90% in most published literature (1). However, some authors have observed a proportion of FL lacking *t*(14;18) as high as 50% in their series, suggesting the existence of marked geographical differences and alternative mechanisms of genetic deregulation in *BCL2*- cases. It is true that some cytogenetic changes may be missed by FISH analysis, such as translocations between *BCL2* gene and unusual partner or cryptic translocations not detected by commercially available probes, however this represents a rare occurrence [(50, 51), personal unpublished data]. Reported detection rates are significantly lower in Far East and, to a less extent, European studies, compared to United States series [(52, 53), personal unpublished data]. Interestingly, in our FISH analysis of a Northern Italian series of FL, we tested different commercially available probes for *BCL2* translocation, obtaining overlapping results in terms of lower percentage of translocated cases than expected, with the best performance obtained using break-apart FISH strategy on formalin fixed and paraffin embedded sections, in analogy with the results obtained on diffuse large B-cell lymphoma (54). When we looked at the different probes design, we found that they were very similar in terms both of DNA size and positional mapping. In addition, as we had also the opportunity of performing karyotype analyses on a consistent number of our cases, we demonstrated that chromosome abnormalities other that *t*(14;18) could characterize follicular lymphomas (manuscript in preparation). As a whole, these observations tend to confirm that the variation in the incidence of *t*(14;18) across studies is due to geographical gradient rather than technical problems, as suggested by others (55, 56). In fact, the relative lower incidence of FL registered in Asian populations seems not to correspond to a lower frequency of *BCL2* rearrangements in healthy individuals (52). It is conceivable that distinct pathogenetic pathways operating in different geographic regions really exist, resulting in FLs that are morphologically similar but molecularly distinct. In this regard, epidemiological data derived from Asian emigrants to the United States and their descendants seem to imply environmental rather than genetic influences (57). For example, cigarette smoke and pesticide exposure have been called into question (52), but the precise nature of such putative factors is far from being elucidated.

The morphological, immunohistochemical, and genetic profiles of grade 3A FLs still resemble low grade disease, whereas FL 3B represents an enigmatic entity with peculiar features, more closely related to DLBCL than to other FLs (58–61). Herein we limit our considerations to classical nodal grade 1–3A FLs, but it is important to keep in mind that several studies addressing the pathogenesis of *BCL2*-negative FL do not specifically exclude pure FL 3B or even DLBCL with an additional FL 3B component from their series, introducing an important confounding factor. It is well-established that nodal grade 1–3A FLs without *t*(14;18) are morphologically indistinguishable from their translocated counterpart, but they are variably characterized by weak or loss of CD10 expression, increased Ki-67 labeling, higher MUM1 and granzyme B immunoreactivity and occasional CD23 positivity in lymphoma cells (62). Moreover, this subset revealed a characteristic miRNA expression profile indicating a late GC B cell phenotype (63). Accordingly, GEP analyses documented an enrichment of GC B cell associated signatures in *t*(14;18)+ FL, whereas ABC-like, NFkB, proliferation, and bystander cell signatures were enriched in negative cases. According to clinical parameters, patients with *t*(14;18)-negative FL had more frequently lower stage disease, even though without any significant impact on overall survival (62). These findings demonstrate distinct molecular features between the two subsets, however, they do not shed light on the molecular pathogenesis of *t*(14;18)-negative FL. At CGH and high resolution single nucleotide polymorphism analyses no alterations that were specific for *t*(14;18)-negative FLs emerged and, importantly, the frequency of *BCL6* rearrangements did not significantly differ between the 2 groups (62).

*BCL6* gene encodes a transcriptional repressor whose oncogenic effect is well-recognized (64, 65). 3q27/*BCL6* rearrangement has been variously reported as a transforming and proliferating stimulus alternative to the classic *BCL2* deregulation in high grade FL (55, 66) or in low grade disease (67). Others again have challenged its putative role as a crucial pathogenetic factor in *BCL2-*negative FL, suggesting that *BCL6* amplification/3q27 gain itself is associated with peculiar clinicopathologic characteristics, namely, high grade morphology, high BCL2 and MUM1 protein expression and frequent combination with *BCL2* gene amplification/18q21 gain (68). In this regard, it is important to remember that BCL2 protein overexpression has been recorded in a subset of FL lacking *t*(14;18) [(60, 69), personal unpublished data]. Many of them showed extra copies of chromosome 18, which may implicate an increased dosage effect, and alternative mechanisms may operate in the remaining unamplified cases (60).

In an attempt to identify pro-survival signals alternative to BCL2 overexpression, increased levels of some proteins such as Bcl-XL and activation of Akt/Bad pathway have been preferentially described in *t*(14;18)-negative FL (70, 71). Moreover, high expression of BCL6 or p53 with a significant inverse relationship between them was reported (72). These data suggest that nodal FL represents a single disease caused by disruption at various levels of a common biochemical pathway, however no convincing deregulation of anti-apoptotic proteins alternative to BCL2 has been demonstrated so far. As already stated, SHM process of *IG* genes is a characteristic feature of FL generating intraclonal heterogeneity. In a small series of 2 and 3A FL, Gagyi and coworkers found no differences in terms of ongoing SHM of the *IGVH* genes, aberrant SHM and AID expression between cases without *BCL2* gene rearrangement and protein expression and lymphomas carrying the *t*(14;18). The authors hypothesize that, besides different molecular alterations at the starting point of lymphomagenesis, *BCL2*-positive and *BCL2*-negative FL represent the same entity sharing several molecular pathways, as in both cases the immunoglobulin receptor complex provides additional signals required for malignant transformation (73). Katzenberger and coworkers identified a distinctive subtype of *t*(14;18)-negative FL, characterized by a predominantly diffuse growth pattern, localized involvement of inguinal lymph nodes and 1p36 deletion (74). Aberrations of this chromosomal region have been reported in *BCL2-* morphologically classical FL with a predominantly follicular growth pattern, but it should be noted that they represent one of the most common alteration in classical *BCL2*+ FL too [(17, 75),personal unpublished data].

In conclusion, to date the genetic events operating within the GC microenvironment and triggering *t*(14;18)-negative FL pathogenesis still remain to be defined.

## Closing Remarks

Follicular lymphoma (FL) is historically considered a well-defined disease, with straightforward diagnostic criteria, a clear genetic background and recognizable precursor condition. In the last few years, a deeper insight in the clinicopathological features of FL has unveiled that this disease is, in fact, composed of many different entities, which raised the need of personalized diagnostic and therapeutic approaches. In this scenario, the genetic landscape of FL, investigated with new technologies, has proved to be variegated and complex. The awareness of the genetic heterogeneity of FL may help finding new treatment strategies for the optimal management of the patients.

## Author Contributions

FM performed the literature review and wrote the manuscript. MT discussed the data and the design of the manuscript, and approved its final version. SU designed the study, wrote the manuscript, and revised the final version.

### Conflict of Interest Statement

The authors declare that the research was conducted in the absence of any commercial or financial relationships that could be construed as a potential conflict of interest.
